# Double-Gated Nanohelix as a Novel Tunable Binary Superlattice

**DOI:** 10.1186/s11671-019-3069-9

**Published:** 2019-08-26

**Authors:** Thomas P. Collier, Mikhail E. Portnoi

**Affiliations:** 1 School of Physics, University of Exeter, Stocker Road, Exeter, EX4 4QL United Kingdom; 2 ITMO University, St. Petersburg, 197101 Russia

**Keywords:** Nanohelix, Non-simply-connected nanostructures, Superlattice, Binary superlattice, Energy bands crossings

## Abstract

We theoretically investigate the problem of an electron confined to a nanohelix between two parallel gates modelled as charged wires. The double-gated nanohelix system is a binary superlattice with properties highly sensitive to the gate voltages. In particular, the band structure exhibits energy band crossings for certain combinations of gate voltages, which could lead to quasi-relativistic Dirac-like phenomena. Our analysis for optical transitions induced by linearly and circularly polarized light suggests that a double-gated nanohelix can be used for versatile optoelectronic applications.

## Introduction

From the fossilized spiralling gastropods that the first author enthusiastically collected in his childhood, to the entwined structure of DNA which undoubtedly once defined those prehistoric creatures, the helix geometry is prevalent throughout nature [1]. Inspired by the complex functionalities attributed to the shapes of naturally occuring bio-molecules [2–6], it is expected that other systems possessing helical geometries suitable for nanotechnology will yield rich physics and contribute to novel applications. Over the past three decades, remarkable progress in nano-fabrication techniques has led to the realization of nanohelices in a host of different systems including InGaAs/GaAs [7], Si/SiGe [8], ZnO [9–11], CdS [12], SiO_2_/SiC [13, 14], and pure carbon [15–20], as well as II-VI and III-V semiconductors [21] (for the current state of the art see Refs. [21–26]). Consequently, a plethora of phenomena is expected in such structures ranging from exotic transport properties like topological quantized charge pumping [27, 28], superconductivity [29], and spin filtering [30–32], to molecular and nanomechanical stretchable electronics [33, 34] due to piezoelectric effects [35], sensing applications [36, 37], energy- [38] and hydrogen-storage [39], and field-effect transistors [40, 41].

The fascination in nanohelix-based devices ultimately stems from the inherent periodicity encoded in the topology of the helix structure. In particular, subjecting a nanohelix to a transverse electric field (normal to the helix axis) gives rise to superlattice behaviour such as Bragg scattering of electrons on a super-periodic potential, leading to an energy splitting at the edge of the superlattice Brillouin zone between the lowest states linearly tunable by the electric field [42, 43]. This behaviour may result in Bloch oscillations and negative differential conductance [44, 45], and can emphasize spin-polarized transport through helices [31, 46], as well as yield a circular dichroism enhancement useful in nanophotonic chiroptical applications [47]. This system constitutes a unary superlattice and further opens the possibility to use nanohelices as either tunnel diodes or Gunn diodes for frequency multiplying, amplification, and generation or absorption of radiation in the eulogized terahertz range [48–51]. While the prototypical superlattice is usually realized in heterostructures of alternating semiconductor layers with different intrinsic band gaps, the parameters of the nanohelix superlattice are fully controlled by the external field. Contrarily, the shapes of the former conventional superlattice potentials are specific to the heterostructure and, while robust, offer limited ability for manipulation in the course of its exploitation without the use of large external fields. Therefore, the appeal in using nanohelices as superlattices in lieu of this lies in their greater tunability.

On the other hand, with heterostructure semiconducting superlattices (or indeed photonic superlattice structures [52–55] and cold atoms in optical lattices [56, 57]) one can create more complicated superlattice unit cells beyond the simple quantum well which is induced by the electric field along the helix. Even the extension to a binary superlattice [58–60] (whereby the unit cell is distinguished by two differing quantum wells and/or barriers) promises a rich array of physics such as Bloch-Zener oscillations [61], which may in turn contribute to tunable beam splitter and interferometer applications [62]. Thus, it would be highly desirable to combine the external-field-tunability of a nanohelix-based superlattice with the superior functionality of a binary superlattice.

In what follows, we describe just such a system, with a nanohelix positioned between two parallel-gated charged wires aligned with the helix axis. We envisage the application of an additional transverse electric field and theoretically show that the gate- and field-controllable potential constitutes a binary superlattice along the one-dimensional helix.

## Methods

### Theoretical model

**Fig. 1:**
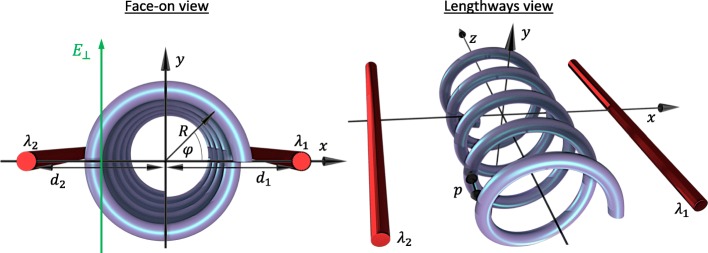
Diagram of the system’s geometry and parameters from both face-on and lengthways perspectives. *R* is the helix radius, and *d*_1_ and *d*_2_ are the distances of the charged wires from the helix axis with charge densities *λ*_1_ and *λ*_2_, respectively. The spatial coordinate *φ* describes the angular position on the helix from face-on and is related to the *z*-coordinate via *φ*=2*πz*/*p* with *p* the helix’s pitch. A transverse electric field *E*_⊥_ is applied parallel to the *y*-axis

Let us start by studying the case of a single-electron semiconductor circular nanohelix with *N* turns of radius *R*, pitch *p*, and total length *L*=*Np*. The nanostructure is positioned between two parallel gates modelled as charged wires with its helix axis aligned along the *z*-axis and with axis and gates all residing on the same plane as depicted in Fig. 1. Additionally, we consider an external transverse electric field normal to the gate-axis plane $\mathbf {E}=E_{\bot } \hat {\mathbf {y}}$ which can be used to break the reflection symmetry of the potential above the plane with respect to the potential below the plane. We work in helical coordinates parametrically described via **r**=(*x,y*,*z*)=(*R* cos(*sφ*),*R* sin(*sφ*),*ρφ*), where the dynamical angular coordinate *φ*=*z*/*ρ* depends only on the distance along the axis of the helix with *ρ*=*p*/2*π*, and *s*=±1 indicates a left- or right-handed helix, respectively. In this work, we consider a left-handed helix *s*=1. In the framework of the effective mass model, the energy spectrum *ε*_*ν*_ of the *ν*th eigenstate of an electron in a helix under the influence of such external potentials is found from the Schrödinger equation:

1 $$ -\thinspace \frac{\hbar^{2}}{2M^{*}\rho^{2}}\frac{d^{2}}{d\varphi^{2}}\psi_{\nu} +\left[V_{g} (\varphi) + V_{\bot} (\varphi) \right]\psi_{\nu} = \varepsilon_{\nu} \psi_{\nu}   $$

where we have geometrically renormalized the electron effective mass *M*_*e*_ to *M*^∗^=*M*_*e*_(1+*R*^2^/*ρ*^2^) in order to express everything in terms of the coordinate along the helix axis (recall that *φ*=*z*/*ρ*) which is more convenient for external potentials. Here, *V*_⊥_(*φ*)=−*eE*_⊥_*R* sin(*φ*) is the contribution from the transverse electric field directed along the *y*-axis such that *V*_⊥_(*π*/2)<0. The potential from the gates is *V*_*g*_(*φ*)=−*e*[*Φ*_1_(*φ*)+*Φ*_2_(*φ*)] with the electrostatic potential felt by an electron along the helix due to an individual charged wire given by *Φ*_*i*_(*φ*)=−*λ*_*i*_*k* ln(*r*_*i*_/*d*_*i*_). Here, *i*=1,2 labels the wires, *λ*_*i*_ is the linear charge density on a wire, and $k = 1/2\pi \tilde {\epsilon }$ with $\tilde {\epsilon }$ the absolute permittivity. The perpendicular distance of a test charge from a particular wire is given by $r_{i}=[d^{2}_{i}+R^{2} + 2(-1)^{i}d_{i}R\cos (\varphi)]^{1/2}$, with *d*_*i*_ denoting the corresponding distance of the wire to the axis of the helix. We have defined zero gate-induced potential to be along the axis of the helix. The total one-dimensional potential *V*_*T*_(*φ*)=*V*_*g*_(*φ*)+*V*_⊥_(*φ*) is clearly periodic *V*_*T*_(*φ*)=*V*_*T*_(*φ*+2*πn*) with period 2*π* in general (which corresponds to period of *p* with respect to the coordinate *z*). This period is significantly larger than the interatomic distance and gives rise to typical superlattice effects. This letter differs from a nanohelix in a transverse electric field (which can be reproduced with *V*_*T*_(*φ*)=*V*_⊥_(*φ*) here) principally by manipulating the repeated unit cell of the superlattice via the double-gate potential *V*_*g*_(*φ*). Taking the limit *p*→0, we return to the particle on a ring picture subject to two electrostatic gates [63, 64]. Making the approximation *R*/*d*_*i*_≪1, we may expand *V*_*g*_(*φ*) up to second order in cos(*φ*), and upon transforming Eq. 1 into dimensionless form we come to

2 $$ {\begin{aligned} \frac{d^{2} \psi_{\nu}}{d\varphi^{2}}+\left[\epsilon_{\nu} + 2A_{g}\cos(\varphi) + 2B_{g}\cos(2\varphi) + 2C_{\bot} \sin(\varphi) \right]\psi_{\nu} = 0,  \end{aligned}}  $$

with the quantities in units of the energy scale $\varepsilon _{0}(\rho) = \hbar ^{2} / 2 M^{*} \rho ^{2}$ defined as

3 $$\begin{array}{@{}rcl@{}} A_{g} &=& \beta\frac{\left(d_{1}^{2} +R^{2}\right)}{d_{1} R}(1-\gamma), \qquad B_{g} = \frac{\beta}{2}\left(1+\frac{\lambda_{1}}{\lambda_{2}}\gamma^{2}\right), \\ C_{\bot} &=& e E_{\bot} R /2\varepsilon_{0}(\rho), \qquad \qquad \qquad \; \epsilon_{\nu} =\frac{\varepsilon_{\nu}}{\varepsilon_{0}(\rho)}. \end{array} $$

**Fig. 2:**
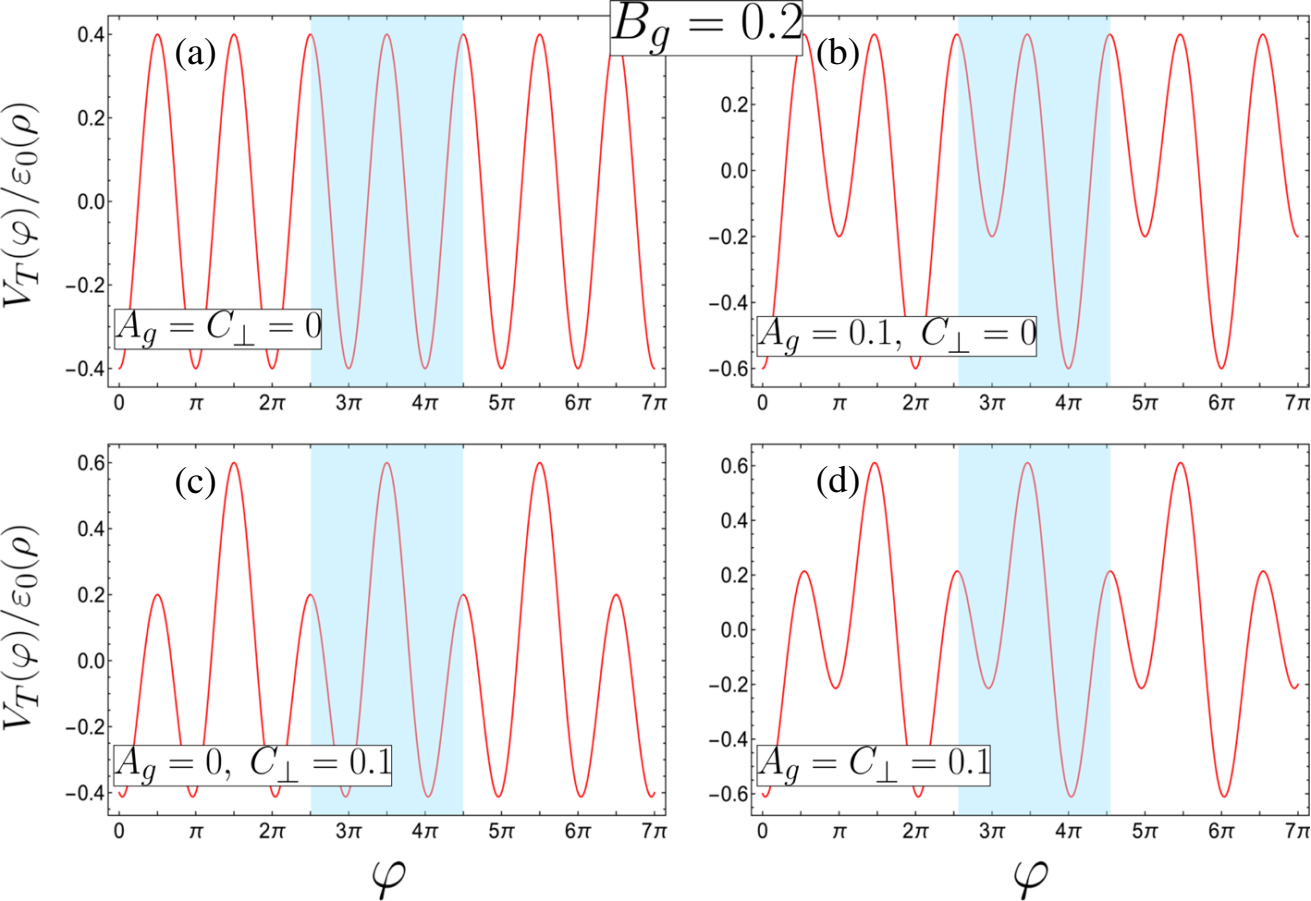
The four possible superlattice potential configurations with the unit cells highlighted in blue (defined in terms of the dimensionless parameters, see Eq. 3 for the corresponding requirements of the physical parameters, and all with *B*_*g*_=0.2). **a** A unary superlattice with degenerate minima and maxima in the unit cell (*A*_*g*_=*C*_⊥_=0). **b**–**d** Binary superlattices formed from either **b** an asymmetric DQW with differing minima and internal reflection symmetry about either minima due to degenerate maxima (*A*_*g*_=0.1, *C*_⊥_=0), **c** a symmetric DQW with degenerate minima only (*A*_*g*_=0, *C*_⊥_=0.1), or **d** an asymmetric DQW with differing minima and maxima (*A*_*g*_=*C*_⊥_=0.1)

Here, $\beta = e k d_{1}^{2} R^{2} \lambda _{1} /2\left (d_{1}^{2} +R^{2}\right)^{2}\varepsilon _{0}(\rho) $ characterizes the contribution from gate 1 while the asymmetry parameter $\gamma =\lambda _{2} d_{2} \left (d_{1}^{2} +R^{2}\right)/\lambda _{1} d_{1} \left (d_{2}^{2} + R^{2}\right)$ characterizes the relative contribution from gate 2, with *γ*=1 corresponding to equal gate contributions to the potential (resulting in *A*_*g*_=0). It should be noted that the inevitable asymmetry caused by the difficulty in maintaining *d*_1_=*d*_2_ can be compensated by manipulating *λ*_1_ and *λ*_2_. In this letter, we restrict ourselves to considering *γ*≤1 (which is |*Φ*_1_|>|*Φ*_2_|) as the asymmetry parameter being greater than unity can be mapped to an equivalent system below unity via a simple exchange of the indices labelling the gates and corresponding shift in perspective *φ*→*φ*±*π*. We will also only consider *C*_⊥_≥0 due to the symmetry of negative *C*_⊥_ with respect to such a coordinate translation in *φ*, and *A*_*g*_≥0, *B*_*g*_>0 (i.e. only positive charge densities on the wires *β*>0) as any potential landscape with negatively charged gates can be reproduced with the correct combination of parameters from positively charged gates. In Fig. 2, we plot the dimensionless potential *V*_*T*_(*φ*)/*ε*_0_(*ρ*), with the strength of the *π*-periodic potential component fixed at *B*_*g*_=0.2, for several combinations of the doubled period perturbation parameters *A*_*g*_ and *C*_⊥_. We see that the total external potential induces a binary superlattice along *φ*, with a double quantum well (DQW) as a unit cell highlighted in blue. This can take qualitatively different forms by manipulating the relative gate contributions *γ* and transverse electric field *E*_⊥_. The unit cell is essentially a single-well under equivalent gate contributions (*γ*=1) and no transverse electric field *E*_⊥_=0 (as in Fig. 2a for *A*_*g*_=*C*_⊥_=0). Fixing *E*_⊥_=0, with a stronger gate 1 contribution (*γ*<1), the unit cell becomes a DQW with differing well minima and degenerate barrier maxima (Fig. 2b where *A*_*g*_=0.1 and *C*_⊥_=0). In contrast, keeping the DQW minima degenerate and manipulating the two potential barriers with respect to each other requires symmetric gate contributions (*γ*=1) in a non-zero electric field *E*_⊥_≠0 (Fig. 2c with *A*_*g*_=0 and *C*_⊥_=0.1). Combining asymmetric gate contributions (*γ*<1) with *E*_⊥_≠0 produces a DQW with differing potential well minima and differing barriers (as seen in Fig. 2d where both *A*_*g*_=*C*_⊥_=0.1). This leads to qualitatively different and rich behaviour as we shall see in the following sections.

### Solutions as an infinite matrix

Solutions to Eq. 2 can be found in terms of the Bloch functions

4 $$ \psi_{n,q}(\varphi)=(2\pi N \rho)^{-\frac{1}{2}}e^{i q \varphi}\sum_{m} c^{(n)}_{m,q} e^{i m \varphi},   $$

where the *q*=*k*_*z*_*ρ* is the dimensionless form of the electron’s quasimomentum *k*_*z*_ along the axis of the helix, *n* indicates the subband, and the prefactor arises from normalization in terms of *φ*: $\rho \int _{0}^{2\pi N}|\psi _{n,q}(\varphi)|^{2} d\varphi = 1$. We make use of the orthogonality of the exponential functions by multiplying the resulting expression by $e^{im^{\prime } \varphi }/2\pi $ and integrating with respect to *φ*, where *m*^′^ is an integer, such that we come to an infinite set of simultaneous equations for the coefficients $c^{(n)}_{m,q}$,

5 $$ {\begin{aligned} &\left[(q+m)^{2}-\epsilon_{n}\right]c^{(n)}_{m} - \left(A_{g} - i C_{\bot} \right)c^{(n)}_{m-1} - \left(A_{g} + i C_{\bot} \right)c^{(n)}_{m+1}\\ &\quad- B_{g}\left(c^{(n)}_{m+2}+c^{(n)}_{m-2} \right)=0,  \end{aligned}}  $$

where for clarity the *q*-subscript notation has been dropped, *ε*_*n,q*_≡*ε*_*n*_ and $c_{m}^{(n)}\equiv c_{m,q}^{(n)} $. Equation 5 represents an infinite penta-diagonal matrix wherein it is apparent that the system is periodic in *q*, and we may restrict our considerations to the first Brillouin zone defined by − 1/2≤*q*≤1/2. In the absence of the superlattice potential *A*_*g*_=*B*_*g*_=*C*_⊥_=0, the eigenvalues are then enumerated by *m* given by *ε*_*m*_=(*m*+*q*)^2^ and we recognize *m* to be the angular momentum quantum number associated with a free electron on a helix. We see from Eq. 5 that when *A*_*g*_=*C*_⊥_=0 only states with *Δm*=±2 are mixed, whereas the formation of a DQW unit cell with differing well minima or barriers, achieved via *A*_*g*_≠0 and/or *C*_⊥_≠0, also mixes states with *Δm*=± 1. Interestingly, the system of an electron on a helix under an external transverse potential (which varies across one revolution of the helix) is mathematically equivalent to an electron on a quantum ring pierced by a magnetic field and subject to a potential with the same functional form varying along the angular coordinate; e.g. see Ref. [65–67] or compare for example Refs. [42–45] with [68–70]. For a ring, the role played by *q* here is taken up by the magnetic flux. Hence, exactly the same analysis in this work is applicable to the problem of a double-gated quantum ring [63–66], were the ring to be pierced by a magnetic flux.

Truncating and numerically diagonalizing the matrix corresponding to Eq. 5 provides the *n*th subband eigenenergies *ε*_*n*_ and coefficients $c_{m}^{(n)}$ for each value of *q*. We apply a truncation at |*m*|=10, safe in the knowledge that any increase in matrix size yields no appreciable change in the lowest subbands.

## Results and Discussion

### Double-gated nanohelix band structure

**Fig. 3:**
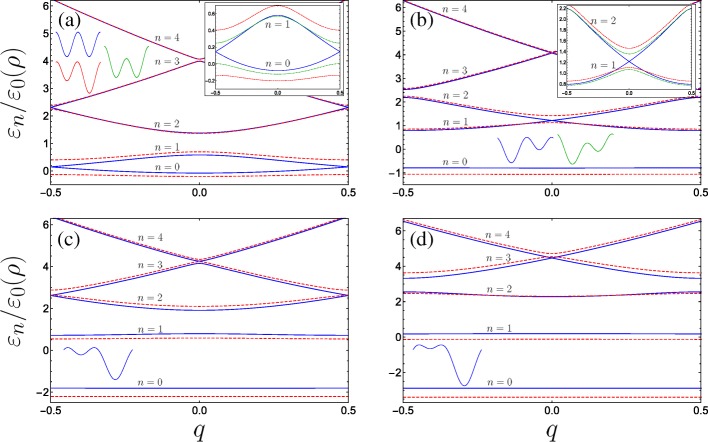
Band structure for double-gated nanohelix system for various combinations of the dimensionless parameters (with *B*_*g*_=0.4 fixed throughout): **a** Solid blue (dashed red) plots *A*_*g*_=0 & *C*_⊥_=0 (*A*_*g*_=0.2 & *C*_⊥_=0), the inset additionally plots the behaviour of the bottom two subbands subject to the transverse electric field with *A*_*g*_=0 & *C*_⊥_=0.2 as the dot-dashed green curve. **b** Solid blue (dashed red) plots *A*_*g*_=0.63 & *C*_⊥_=0 (*A*_*g*_=0.8 & *C*_⊥_=0) where the blue curve depicts the first incident of resonance (see text) with energy bands crossing at the centre of the Brillouin zone, the inset compares the behaviour of the bottom excited two subbands with the case where *A*_*g*_=0.63 & *C*_⊥_=0.2 as the dot-dashed green curve. **c** Solid blue (dashed red) plots *A*_*g*_=1.26 & *C*_⊥_=0 (*A*_*g*_=1.5 & *C*_⊥_=0) where the blue curve depicts the second incident of resonance with energy gaps closing at the edge of the Brillouin for higher bands. **d** The third resonance and higher subband minigaps close at the centre, with solid blue (dashed red) being *A*_*g*_=1.9 & *C*_⊥_=0 (*A*_*g*_=2.2 & *C*_⊥_=0). The unit cell shapes are sketched, *n* enumerates the bands, and the axis of the insets are the same as the main graphs

We plot in Fig. 3 the energy dispersion of the lowest bands for several combinations of parameters. Depending on the form of the superlattice we find a remarkable variety in the dispersion behaviour, and for some specific combinations of parameters we discover energy band crossings for particular subbands at either the edge of the Brillouin zone (Fig. 3a and c) or at the centre of the Brillouin zone (Fig. 3b and d).

#### Low field doubled period perturbation

**Fig. 4:**
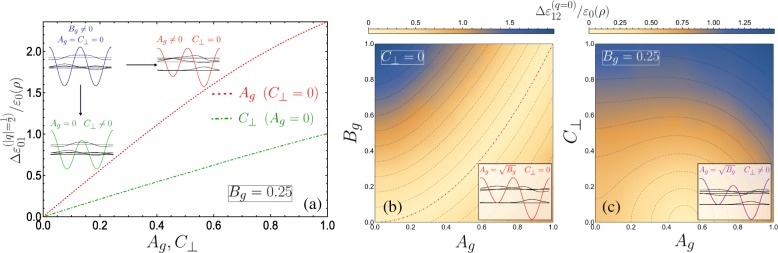
**a** Band gap size between ground and first subbands as a function of *A*_*g*_ (*C*_⊥_) plotted as dashed red (dot-dashed green), here *B*_*g*_=0.25. The diagrams indicate the influence of the different perturbations on the isolated DQW unit cell and eigenstates. **b**–**c** Band gap size between first and second subbands indicated via a 2D density plot as a function of; **b** *A*_*g*_ and *B*_*g*_ for *C*_⊥_=0, and **c** *A*_*g*_ and *C*_⊥_ with fixed *B*_*g*_=0.25. **b** Adjacent isoenergy contour lines indicate a difference of 0.17, with zero gap given by the dot-dashed red line for $A_{g}=\sqrt {B_{g}}$, while **c** the difference is 0.13 with zero gap at the centre of the smallest semi-circle contour (0.5,0). The diagrams sketch the isolated DQW and eigenstates. Hybridization does not occur between the *s*-like and *p*-like resonant localized individual well states in **b**, but does in **c** due to the electric field changing one barrier with respect to the other

When *A*_*g*_=*C*_⊥_=0, the unit cell constitutes two equivalent quantum wells, and consequently the appearance of pairs of bands touching at the Brillouin zone edges arises naturally. Indeed, taking just one well as the unit cell halves the superlattice period and results in a doubling of the Brillouin zone − 1≤*q*≤1. We would then observe the usual unary superlattice band diagram, wherein the band gap between the ground and first bands at *q*=1 is given here via the band gap between *n*=1 and *n*=2 at *q*=0 and would be linear in *B*_*g*_ from perturbation theory. Still, we present a description of the band structure at |*q*|=1/2 in the DQW unit cell picture using matrix algebra in the Appendix. As seen in the inset of Fig. 3a the introduction of either one of the double-period potential terms opens a band gap at the Brillouin zone edge. The unit cell from symmetric gate contributions (*A*_*g*_=0) retains the form of a symmetric DQW under the application of a transverse field *C*_⊥_ perpendicular to the helix-gate axis, with one potential barrier modified with respect to the other (indicated by the green DQW sketch on Fig. 3a). While *C*_⊥_ opens a band gap, the modification of the dispersion is notably less sensitive than to that from a similar magnitude of applied *A*_*g*_. This is seen from the smaller band gap at |*q*|=1/2 for the dot-dashed green line in the inset of Fig. 3a (with *A*_*g*_=0 and *C*_⊥_=0.2) compared to the larger gap for the dashed red curve (which is for *A*_*g*_=0.2 and *C*_⊥_=0). To emphasize this behaviour, Fig. 4a plots the energy gap size at |*q*|=1/2 between the two lowest subbands $\Delta \varepsilon _{01}^{(q=1/2)}/\varepsilon _{0}(\rho)$ for fixed *B*_*g*_=0.25 as a function of both *C*_⊥_ (with *A*_*g*_=0) and *A*_*g*_ (with *C*_⊥_=0), as dot-dashed green and dashed red curves, respectively. In zero transverse electric field and asymmetric gate potentials (*C*_⊥_=0 and *A*_*g*_≠0), the unit cell is then an asymmetric DQW, albeit with internal reflection symmetry about either well minima due to the equivalent barriers. We can then understand the higher sensitivity of the band gap to changing *A*_*g*_ by considering the properties of the isolated DQW unit cell from which the superlattice is constructed. With *A*_*g*_=*C*_⊥_=0, at |*q*|=1/2 (the edges of the Brillouin zone), the Bloch states formed from the ground and first excited state of the isolated DQW unit cell (see the blue schematic and accompanying wave functions in Fig. 4a) will differ only by an arbitrary phase. This situation corresponds to the gapless blue dispersion curve of Fig. 3a. As schematically depicted in Fig. 4a via the green DQW sketch, *C*_⊥_ reduces the relative maxima of one of the barriers with respect to the other, while the DQW minima remain degenerate. As such, the ground state of the isolated DQW is only modified by a slight increase in its probability distribution under the smaller potential barrier (yielding only a small lowering in energy compared to the unperturbed ground state), and the first excited state remains essentially unchanged as its node is positioned under the barrier and is not sensitive to its variation. The Bloch states at the edge of the Brillouin zone which are constructed from these ground and first excited states differ from the unperturbed case only in the reduced decay of the ground state wave function under the smaller barrier (compare the green DQW with the blue DQW in Fig. 4a). Changing *A*_*g*_ manipulates the relative positions of the DQW minima while keeping the barriers degenerate. The wave functions of the two lowest isolated DQW states differ considerably, with the ground state tending towards the localised ground state of the singular deeper well and the first excited state tending towards the localised ground state of the shallower well [71]. While the perturbation lowers the energy of the ground state, the energy of the first excited state is comparatively increased as the minima of the shallower well is shifted up with increasing *A*_*g*_, resulting in the higher sensitivity of the band gap size with respect to *A*_*g*_. In particular, a particle in the ground subband rapidly finds itself confined near the bottom of the deepest potential well with increasing *A*_*g*_. The lowest band therefore approaches a dispersionless flat band swifter than in the transverse field case, which may lead to electronic instabilities and strong interaction effects accompanying the high density of states [72].

#### Energy band crossings

It is quite remarkable to see that if we keep *C*_⊥_=0 and increase *A*_*g*_, while initially all degeneracies are lifted, subsequent higher energy bands are brought to cross each other alternating between the centre and the edge of the Brillouin zone (observe the behaviour of alternating blue and dashed red curves progressing from Fig. 3a through to d). Physically, we can understand the vanishing band gap in terms of interactions of the localized wave functions in the unit cell. When the asymmetric DQW potential is such that the ground state in the shallower well (*s*-like orbital) is resonant with the first excited state in the deeper well (*p*-like orbital), at *q*=0 due to the reflection symmetry about the centre of either well, the opposing parities of these states prevent the usual tunnel-coupling between them, and consequently the excited states constructed from these orbitals coincide (blue curves in Fig. 3b). This is reminiscent of so-called *s*−*p* resonances in optical lattices [73, 74]. By the same token, if the parameters are such that the localized ground state in the shallow well is resonant with an excited state in the deeper well having the same parity, then at |*q*|=1/2, the presence of the Bloch phase fully suppresses the usual hybridization between these two adjacent localized well states and the band gap is closed (as shown in Fig. 3c for resonance of ground with second excited state). In the language of scattering from the periodic potential; the band gap is closed due to the complete destructive interference of the second-order Bragg scattering amplitudes from the cos(*φ*) potential and first-order scattering amplitudes from the cos(2*φ*) potential [75–77].

We can quantitatively show the existence of energy band crossings (for zero transverse electric field) at both the centre and the edge of the Brillouin zone by returning to Eq. 2, which is recognizable as the Whittaker-Hill equation when *C*_⊥_=0 [78]. The Bloch functions Eq. 4 obey twisted periodic boundary conditions *ψ*_*n,q*_(*φ*+2*π*)= exp(2*πiq*)*ψ*_*n,q*_(*φ*). In particular, when *q*=0 formal solutions to Eq. 2 are 2*π*-periodic, whereas when |*q*|=1/2 solutions are 2*π*-antiperiodic (and therefore we shall search for 4*π*-periodic solutions). Specifically, Eq. 2 with *C*_⊥_=0 can be mapped to Ince’s equation [79, 80], which is quasi-exactly solvable, via expressing the wave function as the product of the asymptotic solution to Eq. 2 and an unknown function $\psi _{n,q}(\varphi) = \exp \left [ -2\sqrt {B_{g}}\cos (\varphi)\right ]\Phi _{n,q}(\varphi)$, such that

6 $$ \frac{d^{2} \Phi_{n,q}}{d \varphi^{2}} + \frac{\xi}{2} \sin(\varphi)\frac{d\Phi_{n,q}}{d\varphi} +\frac{1}{4}\left[ \eta_{n,q} - p\xi\cos(\varphi) \right]\Phi_{n,q} = 0,   $$

where we have defined the auxilliary parameters $\xi =8\sqrt {B_{g}}$, *η*_*n,q*_=4*ε*_*n,q*_+8*B*_*g*_, $-p \xi = 8A_{g}+8\sqrt {B_{g}}$, and *Φ*_*n,q*_(*φ*) maintains the necessary twisted periodicity of each solution (note that here *p* is *not* the helix pitch). Additionally, as the superlattice potential here is invariant under the transformation *φ*→− *φ*, the solutions for *q*=0 and *q*=1/2 can be separated into odd and even parity, such that the following trigonometric series

7a $$ \Phi_{n,0}^{(e)}(\varphi) = \sum_{l=0}a^{(n)}_{l}\cos(l\varphi),   $$

7b $$ \Phi_{n,0}^{(o)}(\varphi) = \sum_{l=0}b^{(n)}_{l+1}\sin[(l+1)\varphi],   $$

7c $$ \Phi_{n,\frac{1}{2}}^{(e)}(\varphi) = \sum_{l=0}\widetilde{a}^{(n)}_{l}\cos\left[\left(l+\frac{1}{2}\right)\varphi\right],   $$

7d $$ \Phi_{n,\frac{1}{2}}^{(o)}(\varphi) = \sum_{l=0}\widetilde{b}^{(n)}_{l+1}\sin\left[\left(l+\frac{1}{2}\right)\varphi\right],   $$

cover the formal solutions, and we note that solutions for *q*=−1/2 are the same as for *q*=1/2. Here, the superscripts *e* and *o* label the functions as even and odd, respectively, and *n* still refers to the *n*th subband, which is also the *n*th eigenstate for these specified *q* values. Substituting these into Eq. 6 results in three-term recursion relations for the fourier coefficients. The *q*=0 even solution yields

8a $$ -\eta_{n,0}^{(e)}a^{(n)}_{0} + \xi\left(\frac{p}{2} +1 \right) a^{(n)}_{2} = 0,   $$

8b $$ \xi p a^{(n)}_{0} + \left(4 - \eta_{n,0}^{(e)} \right)a^{(n)}_{2} + \xi \left(\frac{p}{2} +2 \right)a^{(n)}_{4}=0,   $$

8c $$ {\begin{aligned} &\xi \left(\frac{p}{2} - l +1 \right)a^{(n)}_{2l-2} + \left(4l^{2} - \eta_{n,0}^{(e)} \right)a^{(n)}_{2l}\\ &\quad+\xi \left(\frac{p}{2} + l +1 \right)a^{(n)}_{2l+2} = 0, \qquad (l \ge 2)  \end{aligned}}  $$

and the corresponding recursion relations for the odd solution for *q*=0 is

9a $$ (4 - \eta_{n,0}^{(o)})b^{(n)}_{2} + \xi \left(\frac{p}{2} +2 \right)b^{(n)}_{4} = 0,   $$

9b $$ {\begin{aligned} &\xi \left(\frac{p}{2} - l +1 \right)b^{(n)}_{2l-2} + \left(4l^{2} - \eta_{n,0}^{(o)} \right)b^{(n)}_{2l} +\xi \left(\frac{p}{2} + l +1 \right)b^{(n)}_{2l+2}\\ &= 0. \qquad (l \ge 2)  \end{aligned}}  $$

The *q*=1/2 even solution gives

10a $$ \left[ 1 -\eta_{n,\frac{1}{2}}^{(e)} +\frac{\xi}{2}(p+1) \right]\widetilde{a}^{(n)}_{1} +\frac{\xi}{2}(p+3)\widetilde{a}^{(n)}_{3}=0,   $$

10b $$ {}{\begin{aligned} &\frac{\xi}{2}(p-2l +1)\widetilde{a}^{(n)}_{2l-1}+\left[(2l+1)^{2} - \eta_{n,\frac{1}{2}}^{(e)}\right]\widetilde{a}^{(n)}_{2l+1}\\ &\quad+ \frac{\xi}{2}(p+2l+3)\widetilde{a}^{(n)}_{2l+3}=0, \qquad (l \ge 1)  \end{aligned}}  $$

and the *q*=1/2 odd solution gives

11a $$ \left[ 1 -\eta_{n,\frac{1}{2}}^{(e)} -\frac{\xi}{2}(p+1) \right]\widetilde{b}^{(n)}_{1} +\frac{\xi}{2}(p+3)\widetilde{b}^{(n)}_{3}=0   $$

11b $$ {}{\begin{aligned} &\frac{\xi}{2}(p-2l+1)\widetilde{b}^{(n)}_{2l-1}+\left[(2l+1)^{2} -\eta_{n,\frac{1}{2}}^{(e)}\right]\widetilde{b}^{(n)}_{2l+1}\\&\quad + \frac{\xi}{2}(p+2l+3)\widetilde{b}^{(n)}_{2l+3}=0. \qquad (l \ge 1)  \end{aligned}}  $$

Consider then Eqs. (8c) and (9b) for the *q*=0 solutions. The series solutions (7a) and (7b) can clearly be made to terminate if *p* is 0 or an even positive integer. The resulting polynomials are referred to as Ince polynomials. The remaining solutions for higher eigenvalues are simultaneously double degenerate and correspond to the energy crossings observed at *q*=0 for certain parameters. The existence of these degeneracies can be seen by looking at the diagonalizable matrices describing the recursion relations for *a*_*l*_ and *b*_*l*_:

12 $$ \boldsymbol{\mathcal{A}} = \left[ \begin{array}{ccccc} 0 & \xi\left(\frac{p}{2} +1 \right) & 0 & 0 & \hdots \\ \xi p & 4 & \xi\left(\frac{p}{2} +2 \right) & 0 & \hdots \\ 0 & \xi\left(\frac{p}{2} - 1 \right) & 16 & \xi\left(\frac{p}{2} +3 \right) & \hdots \\ \vdots & \vdots & \vdots & \vdots & \ddots \end{array} \right]\!,   $$

and

13 $$ \boldsymbol{\mathcal{B}} = \left[ \begin{array}{ccccc} 4 & \xi\left(\frac{p}{2} +2 \right) & 0 & 0 & \hdots \\ \xi\left(\frac{p}{2} - 1 \right) & 16 & \xi\left(\frac{p}{2} +3 \right) & 0 & \hdots \\ 0 & \xi\left(\frac{p}{2} -2 \right) & 36 & \xi\left(\frac{p}{2} +4 \right) & \hdots \\ \vdots & \vdots & \vdots & \vdots & \ddots \\ \end{array} \right]\!   $$

respectively. Either of the above tridiagonal matrices can be broken into tridiagonal sub-matrices if a leading off-diagonal matrix element is equal to zero, i.e. if *p* is an even number. The matrices will decompose into two tridiagonal blocks, one smaller finite matrix $\boldsymbol {\mathcal {A}_{1}}$ ($\boldsymbol {\mathcal {B}_{1}}$) and a remaining infinite matrix $\boldsymbol {\mathcal {A}_{2}}$ ($\boldsymbol {\mathcal {B}_{2}}$). From the theory of tridiagonal matrices the corresponding eigenvalue spectra for each matrix is then $\eta (\boldsymbol {\mathcal {A}}) = \eta (\boldsymbol {\mathcal {A}_{1}}) \cup \eta (\boldsymbol {\mathcal {A}_{2}})$ and $\eta (\boldsymbol {\mathcal {B}}) = \eta (\boldsymbol {\mathcal {B}_{1}}) \cup \eta (\boldsymbol {\mathcal {B}_{2}})$. The smaller finite matrices are analytically diagonalizable in principle, giving exact eigenvalues, and their corresponding finite length eigenvectors define the fourier coefficients yielding Ince polynomials via Eq. 7. We can see that for a given even integer *p*, the remaining infinite tridiagonal matrices are the same $\boldsymbol {\mathcal {A}_{2}}=\boldsymbol {\mathcal {B}_{2}}\equiv \boldsymbol {\mathcal {D}}$ which results in the double degenerate eigenvalues. To be clear, we provide an example of when *p*=2 in the Appendix.

In the same way, when *p* is a positive odd integer the series solutions (7c) and (7d) can be made to terminate, and the matrices corresponding to $\widetilde {a}_{l}$ and $\widetilde {b}_{l}$ share eigenvalues resulting in the closing of higher subbands at the edge of the Brillouin zone *q*=± 1/2. From the definitions of the auxiliary parameters in Eq. 6, we have

14 $$ A_{g} = (p+1)\sqrt{B_{g}},   $$

which defines the condition for exactly-solvable solutions for the lower lying solutions and simultaneously the existence of higher double degenerate eigenvalues above the *p*th subband, with *p*=0 or an even positive integer corresponding to crossings at the centre of the Brillouin zone, while crossings at the edge require *p* to be an odd positive integer. Figure 4b plots the size of the band gap between the first and second subbands $\Delta \varepsilon _{12}^{(q=0)}/\varepsilon _{0}(\rho)$ as a function of *A*_*g*_ and *B*_*g*_, with the dot-dashed red contour line corresponding to Eq. 14 for *p*=0. The schematic indicates the appropriate eigenstates of the isolated DQW at the *p*=0 resonance.

The application of a small transverse field *C*_⊥_ breaks the reflection symmetry of the system, permitting hybridization of the localized well states of the isolated DQW which results in a significant change at points of degeneracy, as can be seen by comparing the schematic depicted in Fig. 4b with that in c (see also inset of Fig. 3b). We plot in Fig. 4c the behaviour of the band gap between the first and second subbands as a function of *A*_*g*_ and *C*_⊥_. Here we see that the band gap is more sensitive to *C*_⊥_ due to the significant change in the isolated DQW eigenstates by lowering one barrier with respect to the other. This behaviour is notably the converse of the parameter sensitivity for the band gap between the ground and first subbands. By degenerate perturbation theory, it can be shown that this induced band gap is linear in *C*_⊥_ for the lowest crossing bands when *p*=0, and to higher order with increasing *p*. Finally, within the vicinity of the crossings, e.g. for small *q* about *q*=0 in Fig. 3a, the dispersions could be approximated as a quasi-relativistic linear dispersion yielding Dirac-like physics, which could permit superfluiditiy [81] for example. The advantage in using nanohelices lies in introducing such phenomena to portable nanostructure based devices, while also exhibiting unusual responses of the charge carriers to circularly polarized radiation [44, 45, 82–85] (or indeed magnetic fields [86, 87]) due to the helical spatial confinement.

### Optical transitions

In order to understand how our double-gated nanohelix system interacts with electromagnetic radiation, we study the inter-subband momentum operator matrix element $T^{g\rightarrow f}_{j} = \langle {f}|\boldsymbol {\hat {j}} \cdot \boldsymbol {\hat {P}}_{j} |{g}$, which is proportional to the corresponding transition dipole moment, and dictates the transition rate between subbands *ψ*_*f*_ and *ψ*_*g*_. Here, $\boldsymbol {\hat {j}}$ is the projection of the radiation polarization vector onto the coordinate axes (*j*=*x,y*,*z*) and the respective self-adjoint momentum operators are [44, 45, 82–84]

15a $$ \boldsymbol{\hat{P}}_{x} =\boldsymbol{\hat{x}}\frac{i \hbar R}{\rho^{2} +R^{2}}\left[\sin(\varphi)\frac{d}{d\varphi} + \frac{1}{2}\cos(\varphi) \right],   $$

15b $$ \boldsymbol{\hat{P}}_{y}=-\boldsymbol{\hat{y}}\frac{i \hbar R}{\rho^{2} +R^{2}}\left[\cos(\varphi)\frac{d}{d\varphi} - \frac{1}{2}\sin(\varphi) \right],   $$

15c $$ \boldsymbol{\hat{P}}_{z}=-\boldsymbol{\hat{z}}\frac{i \hbar \rho}{\rho^{2} +R^{2}}\frac{d}{d\varphi}.   $$

In terms of the dimensionless position variable *φ*, we are required to evaluate $T^{g\rightarrow f}_{j} = \rho \int _{0}^{2\pi N}\psi _{f}^{\ast } P_{j} \psi _{g} d\varphi $, and upon substituting in from Eq. 4 we find

16a $$ {\begin{aligned} T^{g\rightarrow f}_{x} &= \frac{i \hbar R}{2\left(\rho^{2} + R^{2} \right)}\sum_{m} c_{m}^{\ast (f)} \left[ c_{m-1}^{(g)} \left(q+m-\frac{1}{2}\right)\right.\\ &\quad\left.-c_{m+1}^{(g)} \left(q+m+\frac{1}{2}\right) \right],  \end{aligned}}  $$

16b $$ {\begin{aligned} T^{g\rightarrow f}_{y} &= \frac{\hbar R}{2\left(\rho^{2} + R^{2} \right)}\sum_{m} c_{m}^{\ast (f)} \left[ c_{m-1}^{(g)} \left(q+m-\frac{1}{2}\right)\right.\\& \left.\quad+c_{m+1}^{(g)} \left(q+m+\frac{1}{2}\right) \right],  \end{aligned}}  $$

16c $$ T^{g\rightarrow f}_{z} = \frac{\hbar \rho}{\left(\rho^{2} + R^{2} \right)} \sum_{m} c_{m}^{\ast (f)} c_{m}^{(g)} (q+m).   $$

**Fig. 5:**
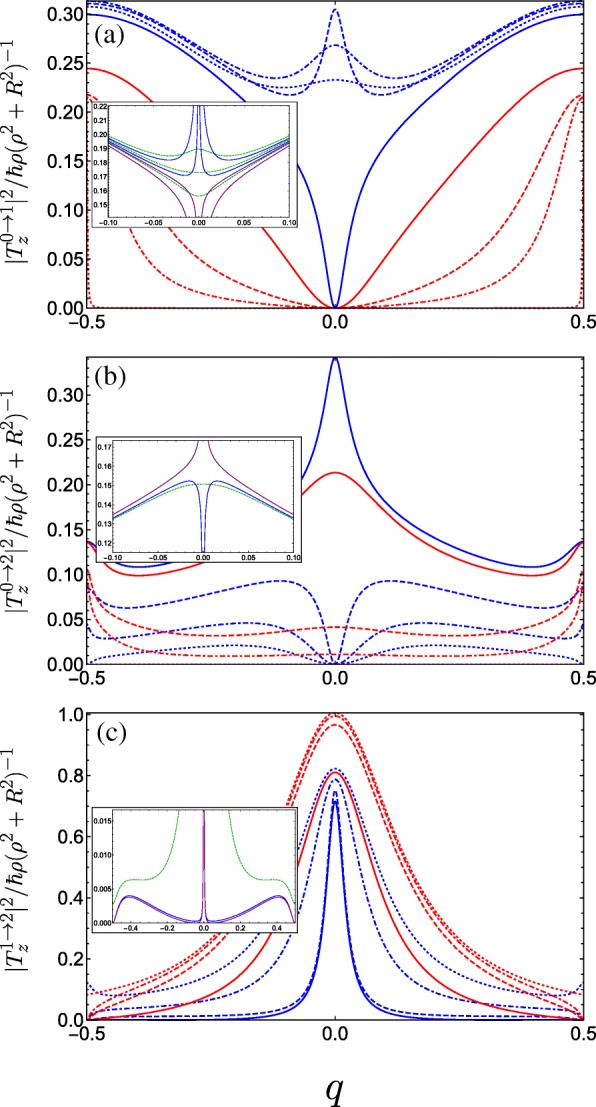
Square of the dimensionless momentum operator matrix element between the *g*th and *f*th subbands in the first Brillouin zone as a function of the dimensionless wave vector *q* of the electrons photoexcited by linearly *z*-polarized radiation and for a variety of parameter combinations spanning the first incident of resonance. The different blue curves keep *A*_*g*_=0.5 and *C*_⊥_=0 fixed and vary *B*_*g*_=0.1, 0.2, and 0.3 corresponding to dot-dashed, dashed, and solid. The different red curves keep *B*_*g*_=0.3 and *C*_⊥_=0 fixed while varying *A*_*g*_=0.05, 0.1 and 0.3 as dot-dashed, dashed, and solid, while the dotted blue (dotted red) plots the limiting case *A*_*g*_=0.5 & *B*_*g*_→0 (*A*_*g*_→0 & *B*_*g*_=0.3). **a** Transitions between the ground and first bands. The inset plots the behaviour for fixed *A*_*g*_=0.5 and changing *B*_*g*_ crossing the resonant condition at *B*_*g*_=0.25 (see text) in a reduced *q*-range, ranging from upper blue *B*_*g*_=0.245, lower blue *B*_*g*_=0.249, upper purple *B*_*g*_=0.251, to lower purple *B*_*g*_=0.255. The dashed green curves are for small non-zero transverse field *C*_⊥_=0.05 ranging from *B*_*g*_=0.245 (upper curve) to *B*_*g*_=0.255 (lower curve) in increments of 0.05. **b** Plots transitions between the ground and second bands, the inset plots the behaviour close to resonance when *A*_*g*_=0.5; blue is *B*_*g*_=0.249, purple is *B*_*g*_=0.251, and dark green is at resonance with *C*_⊥_=0.05. **c** Plots transitions between the first and second bands, the parameters for the inset are the same as those in (**b**)

We see from Eqs. 16a and 16b that light linearly polarized transverse to the helix axis couples coefficients with angular momentum differing by unity *Δm*=± 1, whereas from Eq. 16c, linear polarization parallel to the helix axis couples only *Δm*=0. In Fig. 5, we plot the absolute square of the momentum operator matrix element between the lowest three bands for linearly polarized light propagating perpendicular to the helix axis (i.e. with *z*-polarization). Initially, for *A*_*g*_=*C*_⊥_=0, transitions between the ground and first bands are forbidden (as is to be expected for a unit cell with two equivalent wells resulting in a doubling of the first Brillouin zone, so it is in fact the same band). As the strength of the doubled period potential *A*_*g*_ is increased with respect to *B*_*g*_, transitions become allowed away from *q*=0 as can be seen from Fig. 5a (following behaviour from the dotted red curve through to the solid blue curve). The parameters are swept through a resonance as we go from the solid to the dashed blue curve, wherein the situation changes drastically. To understand this behaviour, we must consider the special case of *q*=0. As we traverse this resonance, the energy of the Bloch function with *q*=0 constructed from the first excited state of the deeper well in the DQW unit cell (*p*-like) passes below the Bloch function constructed from the ground state in the shallower well (*s*-like). Consequently, the parity with respect to *φ* (which is a good quantum number only for *q*=0 or |*q*|=1/2) of the two excited states is exchanged resulting in the rapid switch from forbidden to allowed at *q*=0, wherein the *z*-polarized inter-subband matrix element becomes non-zero due to the operator $\boldsymbol {\hat {P}}_{z}$ (see Eq. 15c) now coupling the even ground state with the odd first excited state. We therefore see the opposite behaviour for transitions between the ground and second band in Fig. 5b about *q*=0. While initially increasing *A*_*g*_ allows transitions at *q*=0 between the ground state and the second excited state when it is *p*-like, beyond resonance (when the order of the *s*-like and *p*-like excited states are swapped) transitions are suppressed. See for example Ref. [88] for a clear picture of this interchange between the ordering of the even and the odd parity excited states. For transitions between the first and second band (Fig. 5c), we observe a large transition centred about *q*=0 due to the lifting of the *m*=± 1 degenerate states of the field-free helix by the superlattice potential. The presence of symmetry-breaking *C*_⊥_ ruins the pristine parities of the states at the centre of the Brillouin zone and all transitions are allowed, as shown in the insets of Fig. 5.

**Fig. 6:**
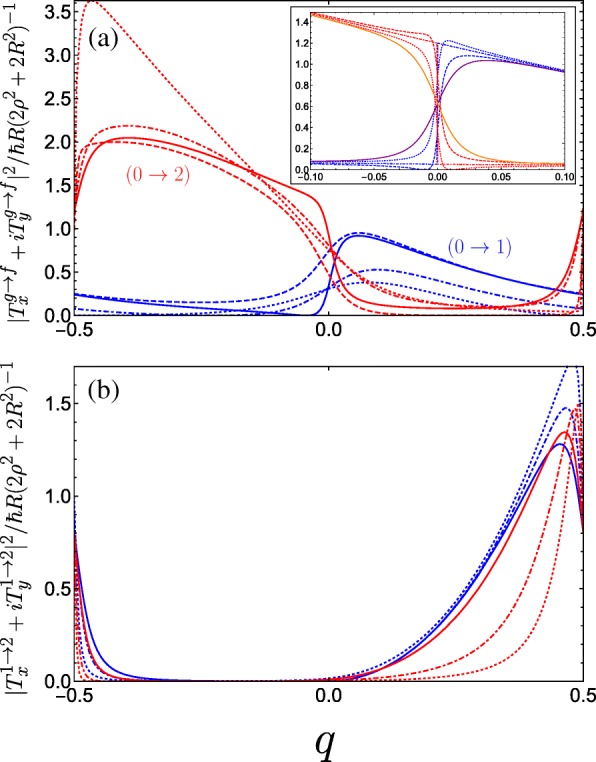
Square of the dimensionless momentum operator matrix element between the *g*th and *f*th subbands in the first Brillouin zone as a function of the dimensionless wave vector *q* of the electrons photoexcited by right-handed circularly polarized radiation |*T*_*x*_+*iT*_*y*_|^2^ and for a variety of parameter combinations spanning the first incident of resonance. **a** The blue curves denote transitions between the ground and first band while the red curves denote transitions between the ground and second band, both with the following parameters: *A*_*g*_=0.5 and *B*_*g*_=0.3 for solid curves, *A*_*g*_=0.5 and *B*_*g*_=0.1 for dashed curves, *A*_*g*_=0.3 and *B*_*g*_=0.3 for dot-dashed curves, and *A*_*g*_=0.01 and *B*_*g*_=0.3 for dotted curves (as *A*_*g*_→0 the maximum of the 0→2 increases rapidly as it approaches *q*=− 1/2). The inset plots the behaviour as *B*_*g*_ is tuned through resonance for *A*_*g*_=0.5; dotted is *B*_*g*_=0.24, dot-dashed is *B*_*g*_=0.25, and dashed is *B*_*g*_=0.26. The solid purple (orange) curve denotes transitions between the ground and first (second) band at resonance with *C*_⊥_=0.05 applied. **b** Plots transitions between the first and second bands. The different blue curves keep *A*_*g*_=0.5 fixed and vary *B*_*g*_=0, 0.2, and 0.3 corresponding to dotted, dot-dashed, and solid. The different red curves keep *B*_*g*_=0.3 fixed while varying *A*_*g*_=0.05, 0.1, and 0.3 as dotted, dot-dashed, and solid. We have omitted plots for *C*_⊥_≠0 here as it yields no great qualitative change to the matrix elements

In Fig. 6, we plot the absolute square of the momentum operator matrix element for right-handed circularly polarized light which propagates along the helix axis, given by |*T*_*x*_+*iT*_*y*_|^2^. Notably, we observe a large anisotropy between the two halves of the first Brillouin zone, while the result for left-handed polarization is a mirror image to what we see in Fig. 6. Physically, this can be attributed to the conversion of the photon angular momenta to the translational motion of the free charge carriers projected onto the direction of the helix axis, with an unequal population of the excited subband in a preferential momentum direction controlled by the relative handedness of both the helix and the circular polarization of light. An intuitive mechanical analogue would be the rotary motion of Archimedes’ screw being converted into the linear motion of water along the direction of the screw axis dictated by the handedness of the thread. As such, our system of a double-gated nanohelix irradiated by circularly polarized light exhibits a photogalvanic effect, whereby one can choose the net direction of current by irradiating with either right- or left-handed circularly polarized light [44, 45, 89]. This differs from conventional one-dimensional superlattices, wherein the circular photogalvanic effect stems from the spin-orbit term appearing in the effective electron Hamiltonian and is consequently a weaker and hard-to-control phenomenon [90, 91]. The electric current induced by promoting electrons from the ground subband to an excited subband *f* via the absorption of circularly polarized light can be understood from the equation for the electric current contribution from the *f*th subband

17 $$ j_{f} = \frac{e}{2 \pi \rho} \int dq \left[ v_{f}(q) \tau_{f}(q) - v_{g}(q) \tau_{g} (q) \right] \Gamma_{CP}^{g \rightarrow f}(q),   $$

where $v_{g,f}(q)=(\rho /\hbar)\partial \varepsilon _{g,f}/\partial q $ is the antisymmetric electron velocity *v*(*q*)=− *v*(− *q*) (which we can deduce from the symmetric dispersion curves), *τ*_*g,f*_(*q*) is a phenomenological relaxation time, and $\Gamma _{CP}^{g \rightarrow f}(q)$ is the transition rate resulting from the optical perturbation of the electron system. Given that $\Gamma _{CP}^{g \rightarrow f}(q) \propto |T_{x}^{g \rightarrow f} + i T_{y}^{g \rightarrow f}|^{2}$ for right-handed circularly polarized light where *T*_*x*_ and *T*_*y*_ are given by Eqs. 16a and 16b, respectively. The anisotropy present in Fig. 6a enters Eq. 17 to yield a non-zero photocurrent. This current flows in the opposite direction for left-handed polarization. Such a circular photogalvanic effect is also exhibited in chiral carbon nanotubes under circularly polarized irradiation [92, 93], although tunability predominantly stems from manipulating the nanotube physical parameters, which are hard to control. The double-gated nanohelix system offers superior versatility by fully controlling the landscape of the superlattice potential, which can be used to tailor the non-equilibrium asymmetric distribution function of photoexcited carriers (as shown in Fig. 6 for inter-subband transitions between the three lowest subbands).

On a side note, we expect that (as with chiral carbon nanotubes [93–95]) the application of a magnetic field along the nanohelix axis can take up the role played by circularly polarized radiation, whereby the current is induced by a magnetic-field-induced asymmetric energy dispersion—which in turn produces an anisotropic electron velocity distribution across the two halves of the Brillouin zone.

## Conclusions

In summary, we have shown that the system of a nanohelix between two aligned gates modelled as charged wires is a tunable binary superlattice. The band structure for this system exhibits a diverse behaviour, in particular revealing energy band crossings accessible via tuning the voltages on the gates. The application of an electric field normal to the plane defined by the gates and the helix axis introduces an additional parameter with which to open a band gap at these crossings. Engineering the band structure *in situ* with the externally induced superlattice potential along a nanohelix provides a clear advantage over conventional heterostructure superlattices with a DQW basis [96, 97]. Both systems can be used as high-responsivity photodetectors, wherein tailoring the band structure (to the so-called band-aligned basis [98–100]) can lead to a reduction in the accompanying dark current. Here control over the global depth of the quantum wells also permits versatility over the detection regime, which can lie within the THz range. We have also investigated the corresponding behaviour of electric dipole transitions between the lowest three subbands induced by both linearly and circularly polarized light, which additionally allows this system to be used for polarization sensitive detection. Finally, the ability to tune the system such that a degenerate excited state is optically accessible from the ground state, along with the inherent chirality present in the light-matter interactions, may make this a promising system for future quantum information processing applications [101]. It is hoped that with the advent of sophisticated nano-fabrication capabilities [102], fully controllable binary superlattice properties will be realized in a nanohelix and will undoubtedly contribute to novel optoelectronic applications.

## Appendix

### Touching energy bands at Brillouin zone boundary when *A*_*g*_=*C*_⊥_=0

Here, we show using matrix algebra that in the picture of a binary superlattice pairs of subbands touch at the Brillouin zone edges if *A*_*g*_=*C*_⊥_=0 and *B*_*g*_≠0, as seen from the solid blue curves in Fig. 3a. Equation 5 is equivalent to the following *N*-by-*N* pentadiagonal matrix Hamiltonian with zeros on the leading sub- and superdiagonals: 
18

Let us consider *q*=1/2 (we could alternatively take *q*=− 1/2) which makes the leading diagonal symmetric. We can then express this matrix Hamiltonian $\widetilde {\mathcal {\boldsymbol {H}}}_{N} \equiv \boldsymbol {\mathcal {H}}_{N,q=1/2} $ in block form as 
19

where 
20

are both of dimension *N*/2-by- *N*/2, and **J** is the exchange matrix. We may construct a matrix via permuting $\boldsymbol {\mathcal {H}}_{N}$ with the *N*-by-*N* permutation matrix $\boldsymbol {\mathcal {P}}_{N}$,

21 $$  \boldsymbol{\mathcal{P}}_{N} = \left[ \begin{array}{cccccc} 1 & 0 & \hdots & \hdots & \hdots & 0 \\ 0 & \hdots & \hdots & \hdots & \hdots & 1 \\ 0 & 1 & \hdots & \hdots & \hdots & 0 \\ 0 & \hdots & \hdots & \hdots & 1 & 0 \\ \vdots & & & & & \vdots \\ 0 & \hdots & \hdots & 1 & \hdots & 0 \\ \end{array} \right],  $$

such that the permutation-similar matrix is 
22

Hence, the eigenvalues of $\boldsymbol {\mathcal {P}}_{N}^{-1}\widetilde {\boldsymbol {\mathcal {H}}}_{N} \boldsymbol {\mathcal {P}}_{N}$, which are the same as the eigenvalues $\widetilde {\boldsymbol {\mathcal {H}}}_{N}$, are double degenerate with the values given by the eigenvalue spectrum of the tridiagonal matrix **C**
23

which can also be expressed succinctly in terms of the previously defined matrices via $\mathbf {C}= \boldsymbol {\mathcal {P}}_{N/2}^{-1}\mathbf {A}\boldsymbol {\mathcal {P}}_{N/2}+\boldsymbol {\mathcal {P}}_{N/2}^{-1}\mathbf {B}\mathbf {J}\boldsymbol {\mathcal {P}}_{N/2}^{4}$. We can see that applying *C*_⊥_≠0 (inset of Fig. 3a) or both *C*_⊥_ and *A*_*g*_≠0 (inset of Fig. 3b) ruins the symmetry in the matrix Hamiltonian and prevents the existence of eigenvalues with multiplicity beyond unity, resulting in the appearance of band gaps.

### Energy crossing at centre of Brillouin zone between third and forth subbands

As an example, let us specifically consider the case where *p*=2, wherein the matrices (12) and (13) become: 
24

and 
25

This case corresponds to the crossings of the blue curves at the edge of the Brillouin zone in Fig. 3d (whereas *p*=0 results in crossings at *q*=0 in Fig. 3b). The lower eigenvalues are found exactly by diagonalizing each of the two finite matrices and they interlace, yielding $\eta _{0,1,2} = 2-\sqrt {4+4\xi ^{2}}, 4, 2+\sqrt {4+4\xi ^{2}}$. The infinite lower-right-hand block tridiagonal matrices coincide, thus the remaining double degenerate eigenvalues are found by approximately or numerically solving Det[***D***−*η****I***]=0.

## Data Availability

The data for the figures all stem from numerically diagonalizing the matrix described by Eq. 5 and can readily be achieved in any numerical software package. With this in mind, the datasets used and/or analyzed during the current study are available from the corresponding author on reasonable request.
